# Assessing the effectiveness of the one paleopathology workshop

**DOI:** 10.1093/emph/eoaf041

**Published:** 2026-01-06

**Authors:** Julianne R Stamer, Mario Apata Mamani, Bernardo Arriaza, Robin Bendrey, Kelly Blevins, Tessa Campbell, Nicole Gottdenker, Rebecca Gowland, Haagen Klaus, Anna Lagia, Judith Littleton, Kirk A Maasch, Carina Marques, Ana Cecilia Mauricio Llonto, Joanna Moore, Elizabeth A Nelson, Lexi O'Donnell, Charlotte Roberts, Daniel H Sandweiss, Ana Luisa Santos, Verena J Schuenemann, Dong Hoon Shin, Thomas Snyder, Anne C Stone, Richard Thomas, Elsa Tomasto-Cagigao, Katherine D Van Schaik, Maricarmen Vega, Joe W Walser, Emily Webster, Jordan A Wilson, Amanda Wissler, Molly Zuckerman, Gwen Robbins Schug, Elizabeth Uhl, Jane E Buikstra

**Affiliations:** School of Human Evolution and Social Change, Arizona State University, 900 Cady Mall, Tempe AZ 85281, USA; School of Human Evolution and Social Change, Arizona State University, 900 Cady Mall, Tempe AZ 85281, USA; Instituto de Alta Invesigación, Universidad de Tarapacá (UTA), Arica, Chile; School of History, Classics and Archaeology, University of Edinburgh, Edinburgh, UK; Department of Archaeology, Durham University, Durham, UK; Institute of Archaeology, University College London, London, UK; College of Veterinary Medicine, University of Georgia, Athens GA 30602, USA; Department of Archaeology, Durham University, Durham, UK; Department of Sociology and Anthropology, George Mason University, Fairfax, VA 22030, USA; Department of Archaeology, Ghent University, Ghent, Belgium; Anthropology, School of Social Sciences, The University of Aukland, Auckland, New Zealand; School of Earth and Climate Sciences, The University of Maine, Orono, ME 04469, USA; Department of Anthropology and School of Integrative Biological and Chemical Sciences, The University of Texas Rio Grande Valley, University Dr, Edinburg TX 78539, USA; Departamento Académico de Humanidades, Pontifical Catholic University of Peru, Lima, Peru; Department of Archaeology, Durham University, Durham, UK; Department of Anthropology, Southern Methodist University, Dallas TX 75205, USA; College of Population Health, University of New Mexico Health Sciences Center, Albuquerque NM 87131, USA; Department of Pathology, University of New Mexico School of Medicine, Albuquerque NM 87131, USA; Department of Anthropology, University of New Mexico, Albuquerque NM 87131, USA; Department of Archaeology, Durham University, Durham, UK (ret); Department of Anthropology, The University of Maine, Orono ME 04469, USA; Department of Life Sciences, Research Centre for Anthropology and Health (CIAS), University of Coimbra, Coimbra, Portugal; Institute of Evolutionary Medicine, University of Basel, Basel, Switzerland; Department of Anatomy and Cell Biology, Seoul National University College of Medicine, Seoul, South Korea; Arts and Science, New York University, New York, NY 10003, US; School of Human Evolution and Social Change, Arizona State University, 900 Cady Mall, Tempe AZ 85281, USA; Archaeology and Ancient History, University of Leicester, Leicester, UK; Departamento Académico de Humanidades, Pontifical Catholic University of Peru, Lima, Peru; Department of Radiology and Radiological Sciences, Vanderbilt University Medical Center, Nashville TN 37232, USA; Departamento Académico de Humanidades, Pontifical Catholic University of Peru, Lima, Peru; National Museum of Iceland, Reykjavik, Iceland; Department of Philosophy, Durham University, Durham, UK; Department of Anthropology, Georgetown University, Washington DC 20057, USA; Department of Anthropology, McMaster University, Hamilton ON, Canada; Department of Anthropology and Middle Eastern Cultures, Mississippi State University, Starkville, MS 39762, USA; Department of Biology, University of North Carolina, Greensboro NC 27412, USA; College of Veterinary Medicine, University of Georgia, Athens GA 30602, USA; School of Human Evolution and Social Change, Arizona State University, 900 Cady Mall, Tempe AZ 85281, USA

**Keywords:** one paleopathology, one health, planetary health, transdisciplinary

## Abstract

**Background and objectives:**

One Paleopathology is a novel concept in Paleopathology that extends the One Health paradigm into the past. A workshop at the University of Durham, UK, was held prior to the 2024 International Society for Evolution, Medicine, and Public Health (ISEMPH) meeting, firstly to define and expand the concept of One Paleopathology and secondly to generate transdisciplinary research and outreach under this framework. This article presents a logic model to evaluate how effectively the workshop met its goals.

**Methodology:**

Two surveys were conducted, one immediately following the workshop and at the 1-year mark. These surveys assess the direct outputs from the workshop—tangible research and outreach products—as well as changes in participants’ attitudes toward One Paleopathology and the degree to which transdisciplinarity was incorporated into resulting projects.

**Results:**

Both the outputs (direct products of the workshop activities) and outcomes (changes in knowledge or attitude because of the activities) of the workshop suggest that the goals are being met. The first goal, to define and expand the concept of One Paleopathology, was met, with participants expressing strong acceptance of the framework. The second goal—generating transdisciplinary research—is reflected in eight ongoing projects initiated at the workshop.

**Conclusions and implications:**

The workshop structure and outcomes assessment presented here evaluate an initial effort in effecting conceptual change in the social sciences. Participants were enthusiastic about One Paleopathology, and over the following year new collaborations and research agendas aligned with the concept emerged. Importantly, participants reported integrating transdisciplinarity into their long-term research, indicating that the workshop had a sustained impact.

## INTRODUCTION

One Paleopathology, which explicitly expands the One Health paradigm that emphasizes the interconnected nature of human, other animals, and the environment in health outcomes [1], to include the past, was introduced in 2022 by Buikstra *et al.* as a way to integrate genomics, non-human animal science, environmental studies, and evolutionary theory within the field of paleopathology [2]. This concept’s premise is a transdisciplinary and multiscale exploration of the past. The One Paleopathology concept embeds humans and non-human animals within diverse ecosystems to link past health dynamics and modern health crises. Through this approach paleopathology becomes deeply relevant for modern biomedical and social challenges such as climate change, health disparities, risk factors in non-communicable diseases, and pandemics. Moreover, it aligns with recent national and global funding priorities on convergence research, which requires ‘merging of ideas, approaches and technologies’ from diverse fields to mitigate current health challenges (e.g. the National Science Foundation’s 10 Big Ideas) [3].

To investigate the broader applications of One Paleopathology and to explore interdisciplinary interest, Buikstra, Robbins, Schug, and Uhl organized a workshop at the University of Durham (UK), held 5–6 August 2024, immediately prior to the annual meeting of the International Society for Evolutionary Medicine and Public Health (ISEMPH). Additionally, during the ISEMPH meeting, One Paleopathology was the focus of a plenary lecture by Buikstra and a symposium on One Paleopathology. The workshop was supported by the National Science Foundation (US), the Center for Bioarchaeological Research (ASU), and the School of Human Evolution and Social Change (ASU). The workshop brought together a transdisciplinary group of researchers with various expertise related to diseases in humans and other animals and environmental health. Envisioned as a model for generating transdisciplinary collaboration, the workshop was built around two goals: (1) to define One Paleopathology as a holistic paradigm, and (2) to generate plans for innovative, convergent, and transdisciplinary (Transdisciplinary research is work that integrates knowledge, skills, methodologies, or perspectives from academic and community partners. Moreover, transdisciplinary work is used to generate outcomes that are transformative and impactful for modern societal challenges [4, 5].) research and outreach.

**Figure 1:**
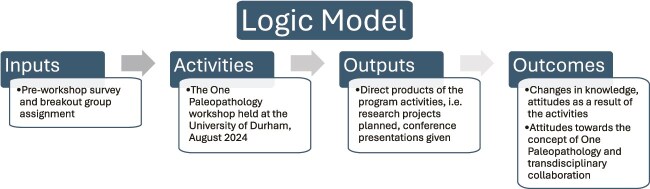
The logic model used for the One Paleopathology workshop with specific goals, we were able to evaluate the outputs (direct products) as well as outcomes (the changes in attitude or knowledge that reflected the accomplishment of those goals.

Here, we will first delineate the concept of One Paleopathology. We then describe events leading up to and during the workshop. Finally, we present our findings regarding the workshop’s effectiveness, following a logic model derived from program theory [6, 7]. Logic models are tools for evaluating specific programs, in this case the One Paleopathology workshop, in relationship to stated goals (Fig. 1 presents the logic model). We thus use a theory-driven model [8] to evaluate the short-term impacts of the workshop. We will describe the inputs and activities of the workshop itself and then, using data from surveys conducted immediately after the event and after 1 year, we describe the direct, tangible outputs of the workshop, such as grant proposals, academic articles, conference presentations, and active research collaborations. We then turn to the outcomes, which include benefits and changes in attitude towards the stated goals [6]. This evaluation method provides a model of change that could be used to assess and refine other scientific workshops to accomplish specific goals.

## ONE PALEOPATHOLOGY

One Paleopathology redefines 21st-century paleopathology [2] as a multi-scale and transdisciplinary framework that extends the One Health paradigm into the past and bridges temporal divides. This approach not only enriches our understanding of historical health dynamics but also delivers actionable insights for current biomedical and public health challenges. For paleopathologists, it integrates veterinary science, genomics (and other -omics), environmental research, and public health data [2, 9]. For researchers beyond Paleopathology, it provides three critical perspectives: (1) long-term assessments of zoonotic and environmental health risks for medical professionals, scientists, and the public; (2) historical models for resilience in the face of ecological crisis and other challenges; and (3) long-term trajectories of human adaptations that contextualize present-day vulnerabilities. These intersections position One Paleopathology as a catalyst for One Heath initiatives [2, 10–12].

One Paleopathology research often addresses more than one of these perspectives, frequently all three simultaneously. The synergy between past and present is exemplified by recent efforts in paleopathology and bioarchaeology to leverage skeletal and archival data to contextualize pandemic responses and the impact of disparities [13]. Among many other insights, paleopathological research on past pandemics—the Black Death or 1918 influenza—reveals that individuals with pre-existing health conditions or physiological stress faced higher mortality risks [14–16]; this pattern is directly relevant to modern health crises like coronavirus disease (COVID-19), wherein frailty guided initial vaccine prioritization. Such work not only informs public health strategies but also engages broader audiences by reframing contemporary challenges through historical analogs. Following the chaos of 2020, when fear and misinformation made implementation of public health measures for COVID-19 difficult, paleopathology helped to depoliticize discussions around vaccination and resilience by grounding them in deep-time evidence [11], offering scholarly and societal value.

One Paleopathology approaches also provides long-term models for the relationships between humans, animals, and their pathogens, as well as how these relationships shift in response to cultural and ecological changes. For example, research on early domesticated goats, which uncovered seasonal brucellosis transmission patterns linked to ancient farming practices [17–20]. These findings do more than reconstruct the past; they directly inform modern public health strategies, from community education campaigns to zoonotic disease prevention frameworks [19–21]. By tracing the enduring relationships between humans, animals, pathogens, and the built environment, such studies exemplify how deep-time scales can simultaneously advance scientific understanding and actionable solutions for One Health challenges. Likewise, such studies highlight how transdisciplinary and deep-time studies provide examples of past human-ecological relationships that can inform future actions.

## INPUTS

**Figure 2:**
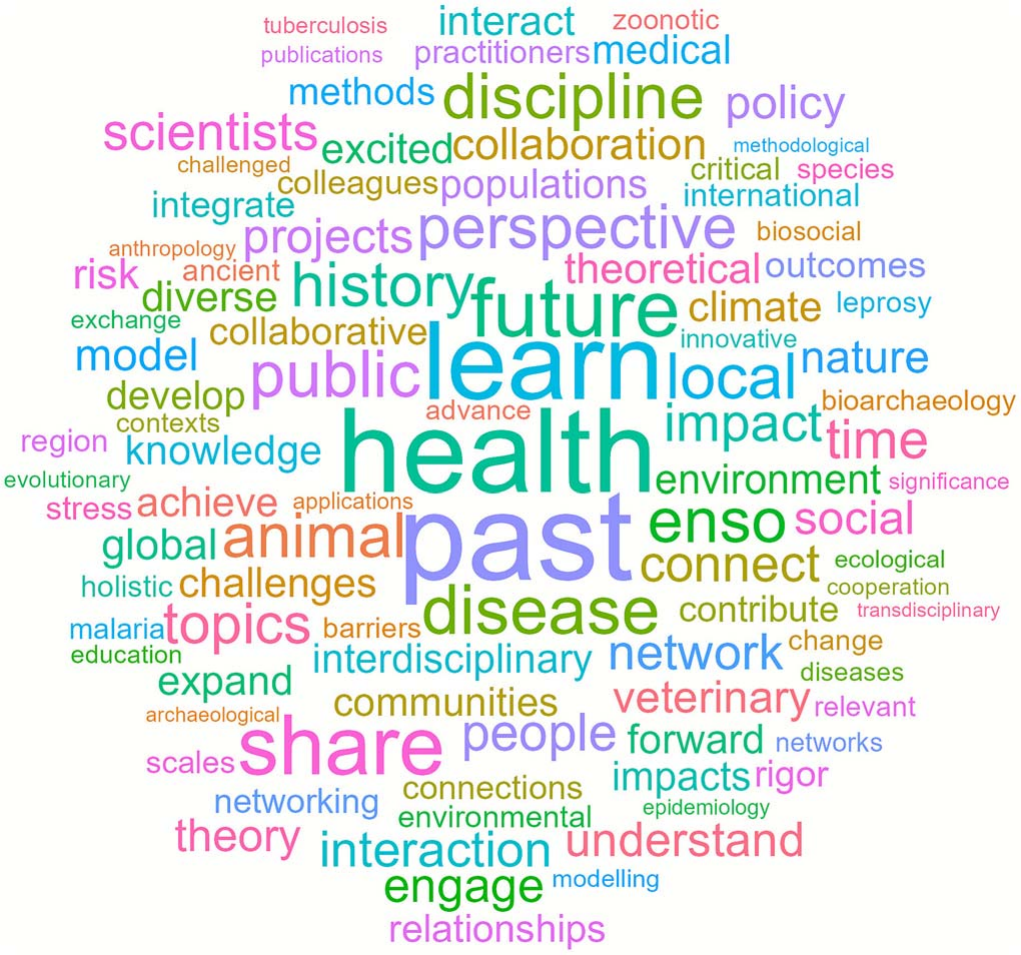
A word cloud generated using tokenized text from the responses of the participants to the pre-workshop survey.

During the months prior to the workshop, we asked participants (*n* = 35) to complete a pre-workshop survey (see Supplementary File A) to gauge topical interest (100% of participants completed this survey). Participants were encouraged to identify one of three pre-defined themes that best aligned with their research interests: disease spillovers, environmental toxicity and health, and climate change. Additionally, they were asked to define their expectations for the workshop as well as potential research questions (within or related to the pre-defined themes) that the participants would like to discuss during the workshop. The organizers provided a synthesis of the survey results, including the potential research questions, to all participants prior to the workshop. To spark discussion and collaboration, we also generated a word cloud from the responses to showcase the variety of topics and ideas that participants mentioned (Fig. 2). We also provided short biographies for each participant, so introductions during the conference would go more smoothly.

We then refined the scope of each major theme using the survey results, identifying distinct research lines. Under the disease spillover category, we created the following working groups: malaria process and risks, animals as disease sentinels (not villains), and syndemics and inequality. Given the breadth of the ‘climate change’ category, we decided to focus on a specific example, the El Niño-Southern Oscillation (ENSO) and the environmental toxicity and health working group was not further divided into topical lines.

We divided participants (*n* = 35) into breakout discussion groups based on participants’ expression of greatest interest. Two participants per discussion group were assigned to facilitate the discussions and note-taking. We paired a more senior researcher with an early-career scholar, a collaboration that appeared to work quite well. Early career scholars were defined as those who had obtained their PhD 8 or fewer years ago and who had not yet attained the rank of tenured Associate Professor. Thirteen participants (37%, *n* = 13/35) were assigned to this category.

## WORKSHOP ACTIVITIES

An international group of thirty-five scholars from the fields of archeology, bioarchaeology, disease ecology, environmental science, history of science and medicine, medical science, climate modeling, paleoparasitology, paleopathology, and veterinary science met at Durham University, UK, for large group sessions and focused breakout discussions over the course of 2 days (see Supplementary File B for the workshop program). On the morning of the first day, a series of presentations outlined the definition and history of One Paleopathology, the goals for the workshop, as well as desired outputs, with emphasis on research projects aligned with National Science Foundation (NSF) program announcements and clearly defined public outreach venues (see Supplementary File B). A second session provided details on rapidly developing methods for imaging analysis using artificial intelligence (AI), genomics and pathology, and ecological modeling. We closed the morning session with five examples of interdisciplinary and transdisciplinary projects, including a historically contextualized case study detailing silver production and the biological and environmental consequences for miners in Peru [22, 23].

During the afternoon of day 1 and morning of day 2, the 35 participants were divided into five breakout groups, apportioned according to pre-workshop survey responses, including (i) malaria process and risks, (ii) animals as disease sentinels (not villains), (iii) syndemics and inequality, (iv) ENSO, (v) environmental toxicity and health. We provided each group with some initial questions (see Supplementary File C) designed to open targeted discussions; the facilitators and discussants were also encouraged to engage in non-guided discussions. In the afternoon of the second day, all participants gathered, and the facilitators shared the main discussion points and outcomes for each breakout group. Along with research and outreach plans.

## OUTPUTS

During the summer of 2025, we surveyed participants about activities that were a direct result of our August 2024 workshop. Specifically, the survey recorded the number of new collaborations and research products, including new grant proposals, academic articles, conference presentations, other forms of research collaboration, and outreach activities that were planned or completed as a result of the workshop. These were deemed outputs and were assessed across one initial survey sent to participants at the end of the workshop, a review of relevant publications and conference presentations from the participants, and a follow-up survey administered to the participants 1 year after the workshop (see Supplementary File D for the post-workshop survey and Supplementary File E for the 1-year post-workshop survey; data provided are the result of individual survey responses, and are provided in the form of counts and frequencies to protect individual responses).

### Post-workshop survey, administered at the end of the workshop

Thirty workshop participants (86%, *n* = 30/35) responded to the first post-workshop survey. This assessment exercise collected information on breakout group participation, plans for collaboration, grant writing, academic writing, and conference preparation, as well as public outreach to be produced. Most (87%, *n* = 26/30) respondents clearly articulated new research collaborations that they had formed during the workshop. Participants reported on the creation of eight new research collaborations with other workshop participants, as well as plans for eight review articles and four outreach-based articles or projects. The environmental toxicity and health group planned one review paper and research project on toxicity in human tissues. They have drafted an outreach paper specifically addressing policymakers. This paper highlights how exposure to environmental toxicity is long-term and provides six policy recommendations for incorporating deep-time archeological data into contemporary regulation on environmental health policy [24]. The ENSO climate group proposed two research projects centered on identifying human responses to ENSO events, as well as a review paper. This group is also writing an invited commentary for Nature Communications on the danger of stating that climate change and stress generate violence [25]. This work provides recommendations for climate and security research as well as policy.

As mentioned above, we divided the disease spillover group into three—animals as sentinels, not villains; malaria; and syndemics and inequality. The Sentinel group planned two review papers to situate the group’s approach within the One Paleopathology paradigm and to review the role of animals in the past. Additionally, this group envisioned an outreach paper to communicate with non-specialist audiences. The malaria group proposed a series of review articles directed at different audiences that would become the basis for grant proposals and a larger project.

### Review of relevant publications

During the year following the workshop, two review articles were produced that provided an overview of the concept of One Paleopathology. The first was a perspective piece published in *BioScience* [16] and the second was an encyclopedia entry for the *Encyclopedia of One Health* [19]. Both review articles targeted specialists in the biomedical sciences. An additional article, discussing the need for a One Health approach to studying animal domestication, was recently published [26]. In addition, the networking opportunities afforded by the workshop have resulted in early career attendees being invited to contribute to pre-existing projects on related topics (e.g. health inequalities).

Several workshops and symposiums have been organized as a direct result of the Durham workshop. First, an invited symposium was held at the 52nd Annual North American Meeting of the Paleopathology Association (2025), which included eight papers on topics ranging from the skeletal signs of lead toxicity in ancient Greece to the relationship between climate change and trauma in Peru. Early career scholars were the lead authors for three presentations (37.5%). Four papers (50%) represented collaborators beyond workshop attendees, demonstrating expanding networks across a wide range of interests. Moreover, an early-career workshop participant co-organized an interdisciplinary workshop focused on syndemics, childhood health, and disease held 4–5 November 2025, at Georgetown University. Looking forward, during the annual meeting of the American Association for the Advancement of Science in 2026, participants from the workshop will participate in a panel discussion entitled ‘Lessons from the Past at Scale: Food Security, Climate Change, and Disease Spillovers.’

### One-year post-workshop survey

Thirty-two workshop participants (91%) responded to a second survey administered ~1 year after the Durham workshop. This survey assessed further developments in research, collaborations, and outreach, based on participants’ self-reporting. To explore intergenerational engagement, participants were sorted into early career and senior scholars.

**Figure 3:**
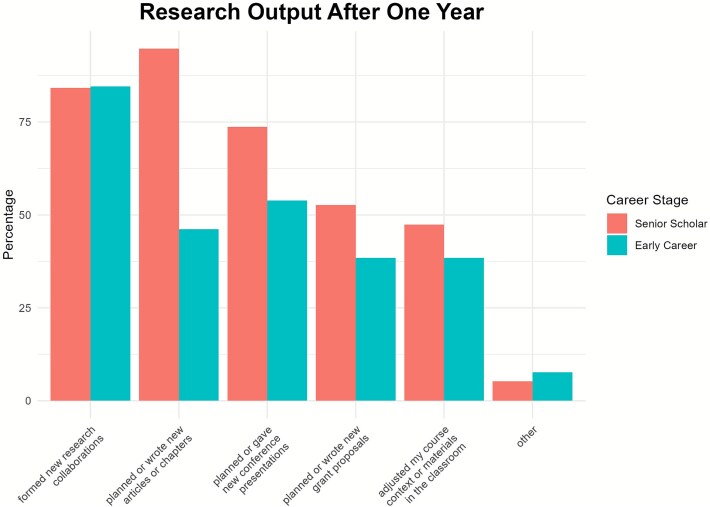
Self-reported research output divided by career stage.

We explicitly asked all participants whether they had engaged in some form of research output, including forming research collaborations, creating written products, presenting conference papers, and adjusting course content. All participants reported at least one research output related to the workshop over the course of the year. Seven individuals (22%) reported having been involved in all five activity categories. Twenty-seven (84%) respondents stated that at the 1-year mark, they had formed new research collaborations due to the workshop, with nearly equal participation by senior (84%, *n* = 16/19) and early career scholars (85%, *n* = 11/13) (see Fig. 3 for a division of responses by career stage). Overall, early career scholars self-reported less participation in research activities than their senior colleagues. However, when asked to name specific results of the workshop, 92% (*n* = 12/13) of early career researchers named a specific collaboration, article, presentation, or research project that involved them, while 79% (*n* = 15/19) of senior scholars did the same.

At the 1-year mark, participants continue to show a vested interest in the One Paleopathology concept. They have reported specific research activities stemming from the workshop. For example, participants of the syndemics working group were inspired to more proactively engage in collaborations with epidemiologists focused on infectious disease risk to explore links between past outbreaks and emerging threats. The continued development of new research by all participants demonstrates both the expansion of the concept of One Paleopathology as well as the transdisciplinarity of One Paleopathology. Future evaluation of workshop outputs will continue to track research activities stemming from the workshop, as well as follow the network of researchers, both participants and non-participants, who contributed to this work.

## OUTCOMES

In addition to polling participants about their research-related outputs from the workshop at the 1-year mark, we also asked how the workshop changed their understanding of the concept of One Paleopathology, contributed to their understanding and engagement with transdisciplinary research, and the benefits of using a One Paleopathology approach.

Nearly all the responding workshop participants (31 of 32; 96%) stated that the One Paleopathology workshop changed their understanding of the One Paleopathology concept. When asked for further discussion, participants mentioned an increased awareness of the need for transdisciplinary work; expansion of their understanding of the relevance of the environment to health in the past; reinforcement of the temporal element that One Paleopathology brings to One Health; and the relevance of studying health in the past to modern health issues. Moreover, when asked to define One Paleopathology, multiple participants stressed the relevance of the concept for contemporary health problems. One stressed the relationship of One Paleopathology to contemporary issues, saying that One Paleopathology was

“… a unifying and action-oriented model that seeks to integrate diverse disciplinary perspectives—such as biological anthropology, archaeology, evolutionary medicine, history, cultural anthropology, genetics, veterinary sciences, and environmental studies—to investigate health [and] disease in past populations in a way that produces knowledge transferable to current challenges and problems. One Paleopathology gives the proper [emphasis] to the collaboration, methodological innovation, and production of knowledge that is applicable to [modern] problems.”

Other participants described human health and disease as ‘interconnected’, ‘integrated’, ‘inextricable’, ‘deeply interwoven’, and ‘unbreakable’ from the holistic relationship to animals, pathogens, and the environment that ‘…goes beyond considering humans as the sole actors.’ Additionally, many of the responses to the questions about defining and expanding the concept of One Paleopathology reported a desire to engage deeply with the One Paleopathology concept and integrate it within pre-existing research frameworks. Moreover, the definitions provided by attendees generally agreed with each other, suggesting a unified understanding of the concept.

However, a few individuals provided reasonable critiques of One Paleopathology or the issues discussed at the workshop. One participant noted that the operationalization of the One Paleopathology concept was still not clearly laid out for both Paleopathology researchers and those from other disciplines. This participant recommended creating materials to clearly outline and operationalize this perspective, including providing a ‘methodological toolkit and guidelines for integrating diverse data about human, animal and environmental data.’ Another participant highlighted the fragmentary nature of the archeological and paleopathological record, emphasizing caution with regard to interpretation based on the limitations of the field, instead suggesting that One Paleopathology ‘be more accurately viewed as contributing a temporal and evolutionary dimension to existing models.’

To better understand the impact of the workshop on how participants viewed the importance of One Paleopathology, they were also asked about their use of the term ‘One Paleopathology’ in future work, as well as their belief that One Paleopathology was a significant concept. Two-thirds (75%) of respondents stated that they would use the term ‘One Paleopathology’, while several respondents believed they would selectively use the term or were more interested in using the term ‘One Health’. One participant wrote ‘[One Paleopathology’s] close association with One Health makes it a succinct term that quickly conveys its interdisciplinary intentions with a foundation in promoting academic collaboration with strong links to educational and public engagement.’ Another participant stated that their direct work with public health professionals made ‘One Health’ a more appropriate word choice, even when they were discussing the applications of One Health concepts in the study of past peoples. Furthermore, many (84%) participants believed that One Paleopathology was a significant concept. One participant stated, with regard to One Paleopathology’s significance for the field of Paleopathology, that ‘Paleopathology needs a more solid theoretical orientation and a stronger articulation of its relevance to contemporary global challenges. We are frequently asked how our work contributes to the present and we often give generalist answers.’ This response reflects the view held by others who emphasized that engagement with contemporary issues is the most significant aspect of One Paleopathology. Moreover, several expressed that they thought One Paleopathology was a necessary paradigm shift for the discipline of Paleopathology, which would make it more relevant to contemporary issues. One such response stated ‘One Paleopathology represents a paradigm shift for the field. It moves beyond diagnostic or purely biomedical models to a systems-based understanding of health in the past (one that recognizes the entanglements of human, animal, and environmental well-being).’

Many (84%) also believed that One Paleopathology was significantly transdisciplinary. Nine (28%) respondents stated that they believed the concept inherently required transdisciplinary research. One participant expressed how the workshop

“expanded my concept of interdisciplinary research by showing the value of actively connecting distinct lines of expertise to build more comprehensive understandings of past health. It emphasized that true interdisciplinary work isn’t just additive, but becomes transformative with cross disciplinary interaction, and that gathering scholars across fields can generate new questions, frameworks, and insights that wouldn’t emerge in disciplinary isolation.”

Nearly half of the respondents (47%) did not believe that their concept or understanding of transdisciplinary research had changed significantly. Several respondents, however, mentioned how the workshop operationalized the way they viewed transdisciplinary research. One participant, already familiar with interdisciplinary work, stated ‘attending the workshop enabled me to recognize that interdisciplinary collaboration can also emerge between fields that are traditionally seen as unrelated. The experience also highlighted for me the importance of pursuing more integrated, transdisciplinary collaborations as a means to advance scientific knowledge.’ However, others expressed a concern for beginning transdisciplinary relationships. While there were many who agreed that the concept of One Paleopathology was transdisciplinary, there were two respondents (6%) who believed that broader collaborative networks are required to accomplish this goal.

## DISCUSSION

### Goal 1: expanding the concept

The first goal of this workshop was to define and expand the breadth of One Paleopathology as a holistic field. Virtually all responding workshop participants stated at the 1-year mark that they believed the workshop changed their understanding of the concept of One Paleopathology and provided specific examples of how the workshop had accomplished this. Moreover, definitions provided by each responding participant for One Paleopathology generally agreed: a unique but unified perspective seems to have emerged. The logic model provides evidence that the first goal is being met at this short-term stage.

### Goal 2: generate transdisciplinary research

The second goal of the workshop was to generate innovative, transdisciplinary research and outreach collaborations. Evidence ranging from clear engagement in research collaboration and contributions to ongoing projects (see Fig. 3) speaks to the generation of research and outreach collaborations by most participants in some capacity. Most participants agreed that transdisciplinary efforts are an important part of One Paleopathology. Some expressed concern over the challenge of being truly interdisciplinary or transdisciplinary, but expressed the desire to continue these efforts. Participants were generally eager to engage with transdisciplinary research and concepts. The workshop was successful in instilling these values and encouraging discussion during the period of this survey. We anticipate that subsequent intermediate- and long-term outcomes will further demonstrate successes in producing transdisciplinary research, stimulated by this workshop.

### Future directions

We present here the short-term outputs and outcomes from this workshop at 1-year post-workshop. To assess intermediate-term and long-term outcomes, the two stated goals will be assessed again at the 5-year mark [7]. In addition to the two stated goals, this study has identified additional outcomes that participants expressed an interest in addressing moving forward.

First, as part of the first stated goal to expand the One Paleopathology concept, several participants expressed an interest in more overt theorizing and operationalization of concepts relating to One Paleopathology, connecting them with broader theory in bioarchaeology and paleopathology [27]. Thus, it is our intention to understand how the concept of One Paleopathology will be used in the future, both by participants from this workshop as well as other scholars. In doing so, we hope to encourage overt theorizing of these concepts.

Second, as part of the second stated goal to generate transdisciplinary research and outreach activities, we aim to better measure the transdisciplinary aspect of the research and outreach activities. Future efforts will reflect on the nature of transdisciplinary research and ask how One Paleopathology is able to be transdisciplinary through an assessment of the types of collaboration and a reflective questionnaire how participants felt that they were transdisciplinary. Workshop organizers will continue to encourage this kind of work moving forward.

Third, we hope to show that One Paleopathology generates meaningful outreach activities, i.e. work with policymakers, and engagement with the public, as intermediate-term and long-term outcomes. At the 1-year mark, there are currently two clear, but nascent, efforts to engage with policymakers. Workshop organizers will continue to encourage these goals, which will be assessed again at the 5-year mark.

Finally, we hope that this workshop’s approach—setting goals and collecting data, measuring how these goals were met—will become a replicable template for other fields of research and other interdisciplinary and transdisciplinary research groups. It may prove transformative for assessing scientific paradigm shifts.

## CONCLUSIONS

The workshop outcomes and review of the activities presented here are short-term. They suggest, however, that the impact of the One Paleopathology workshop will continue over the coming years as the concept continues to be used and expanded. Workshop participants were enthusiastic about the concept, initiating research collaborations and agendas aligned with the One Paleopathology concept, and committing to incorporating transdisciplinarity into their research agenda in the long term.

## Supplementary Material

Supplemental_File_A_-_Pre-Workshop_Survey_eoaf041

Supplemental_File_B_-_Workshop_Program_eoaf041

Supplemental_File_C_-_Pre-workshop_Questions_eoaf041

Supplemental_File_D_-_Post-Workshop_Survey_eoaf041

Supplemental_File_E_-_Outcomes_Assessment_Survey_eoaf041
