# Supplementary material for: Assessing the effectiveness of the one paleopathology workshop
Source: Evol Med Public Health. 2026 Jan 6;14(1):1–10. doi: 10.1093/emph/eoaf041 (PMC12874872; doi:10.1093/emph/eoaf041)
Supplement: Supplemental_File_A_-_Pre-Workshop_Survey_eoaf041 [file supplemental_file_a_-_pre-workshop_survey_eoaf041.pdf]

Pre-Workshop Questionnaire for ONE Paleopathology Conference

Preamble

ONE Health is defined by the US CDC as "*an integrated, unifying approach to the health of people, animals, and ecosystems.*" Recent ONE health research has emphasized the need for research on imminent threats to global human security, including pathogen spillover, climate change, and environmental health. The participants in this workshop will work to define a relatively new research paradigm for ONE Paleopathology, defined as bringing a temporal dimension to contemporary ONE Health research, awareness, and outreach efforts.

This ONE Paleopathology Conference is designed to bring together researchers with special expertise related to diseases in humans and other animals, to include infectious disease transmission, climate change (ENSO case study), environmental health, and other aspects of environmental health. Participants in the workshop include researchers focused on medical history, paleopathology, and ecological modeling to delineate the current state of cross-cutting, potentially transformative research in this area. We will then we will turn to meaningful new approaches and innovative, new questions that will advance bases for research and outreach in the near term. The meeting is also intended to serve as an incubator, facilitating inter-disciplinary interaction and collaboration on these topics.

This workshop links ONE paleopathology with interdisciplinary and innovative themes defined in NSF's 10 Big Ideas initiative, especially Convergence Research[1]. [https://new.nsf.gov/funding/opportunities/growing-convergence-research-gcr/nsf24-527/solicitation?utm_medium=email&utm_source=govdelivery]. Convergence research is defined by two primary characteristics. First, it includes work driven by specific and compelling problems, pressing social concerns, or opportunities to address deep, basic scientific questions. Second, convergence research is based on a premise of deep integration across disciplines. This workshop brings together interdisciplinary experts to apply diverse knowledge, theories, methods, data, research communities and languages to create highly innovative frameworks and to define a transformative paradigm for ONE Paleopathology.

To guide the organizers in their final assignments to breakout groups and in framing specific charges, please answer the following questions.

1. What are your expectations of this workshop? What do you hope to achieve through this interaction?
2. Please identify one (or more) topics of particular interest to you in the realm of:
    a. Pathogen Spillover: history, ecological and environmental correlates, human-animal relations, social determinants of health and syndemics.
    b. Environmental Health Impacts: e.g., superabundance of metals and other environmental toxicants; severe underrepresentation of essential nutrients)

---

[1] The European Union's Horizon Europe initiative may offer similar options for Projects in Europe. https://research-and-innovation.ec.europa.eu/funding/funding-opportunities/funding-programmes-and-open-calls/horizon-europe_en

   c. Climate Change: how have episodic extreme weather events in the past shaped animal and human health and human biocultural diversity (e.g. ENSO); what factors create greater opportunity for resilience in the face of long-term climate shifts or climate variability?
3. Are there other related topics of particular interest to you that we might add to the mix?
4. Provide an example of a currently unanswered question(s) relating to your topic(s) of interest (examples follow), potentially innovative and/or interdisciplinary approaches to the question, and the type of expertise you think would be required to address the question.
5. How will you prepare for the workshop and is there anything that the organizers can provide to help you prepare?
6. Is there anything else we should have asked in this pre-conference questionnaire?

Examples:

- *Burkholderia mallei* and *pseudomallei* cause glanders (obligate pathogen in horses, camels, donkeys) and melioidosis (facultative), respectively. The diseases apparently developed infectivity in Australasia thousands of years ago (Baker et al. 2018) from a freely living bacterium. What conditions were associated with disease transfer between horses and humans? What tools and expertise do we need to address this question and where would be key archaeological sites? (Buikstra & Uhl have a research group working on this topic and would welcome interested colleagues.)
- El Niño events apparently caused coastal Andean peoples to feed higher on the marine food chain (Wilson 2022). How did this change their health? Did increased contact with pinnipeds lead to increased risk of tuberculosis transfer (Bos et al. 2014)? How did the disease landscape for animals and humans change with ENSO events of varying length?
- How can environmental conditions, in particular regional geology, and advances in the pathophysiological understanding of the complex impacts of toxins and nutrient deficiencies on both human and animal health be better incorporated into paleopathological studies?
- How has indigenous knowledge helped animals and humans cope with environmental toxicants and absence of essential nutrients? (Or how have animals and humans adapted to environmental toxicants and absence of essential nutrients?)
- *Mycobacterium lepromatosis, M. leprae,* other MTBC members: their global history and new approaches to understanding zoonotic spillover in the past; co-evolutionary processes with other microorganisms and the microbiome; relationships between spillover/spread and climate/environmental change, historical and sociocultural aspects; and the natural history of disease progression in past populations. What are the important new questions we need to be asking about these pathogens, inter-specific relations, and our co-evolution? What new methods will help us to address intractable questions in this area?
- How can we model ecological correlates of pathogen spillover and spread in relation to "new" (to us) approaches like dental calculus, pathogen genomics, bioinformatics, modeling, Micro CT, and deep learning?

Pre-Workshop Questionnaire

These examples are limited but we hope they provide stimulus for your creative juices. Many of the questions are not new, but we have new tools today (including but not limited to model phylogeographies drawn from contemporary genomics and aDNA, 3-D modalities and Micro CT, bioinformatics and modeling approaches) that should encourage targeted exploration of the archaeological record.
