# Supplementary material for: Assessing the effectiveness of the one paleopathology workshop
Source: Evol Med Public Health. 2026 Jan 6;14(1):1–10. doi: 10.1093/emph/eoaf041 (PMC12874872; doi:10.1093/emph/eoaf041)
Supplement: Supplemental_File_B_-_Workshop_Program_eoaf041 [file supplemental_file_b_-_workshop_program_eoaf041.pdf]

ONE Paleopathology Workshop

Schedule

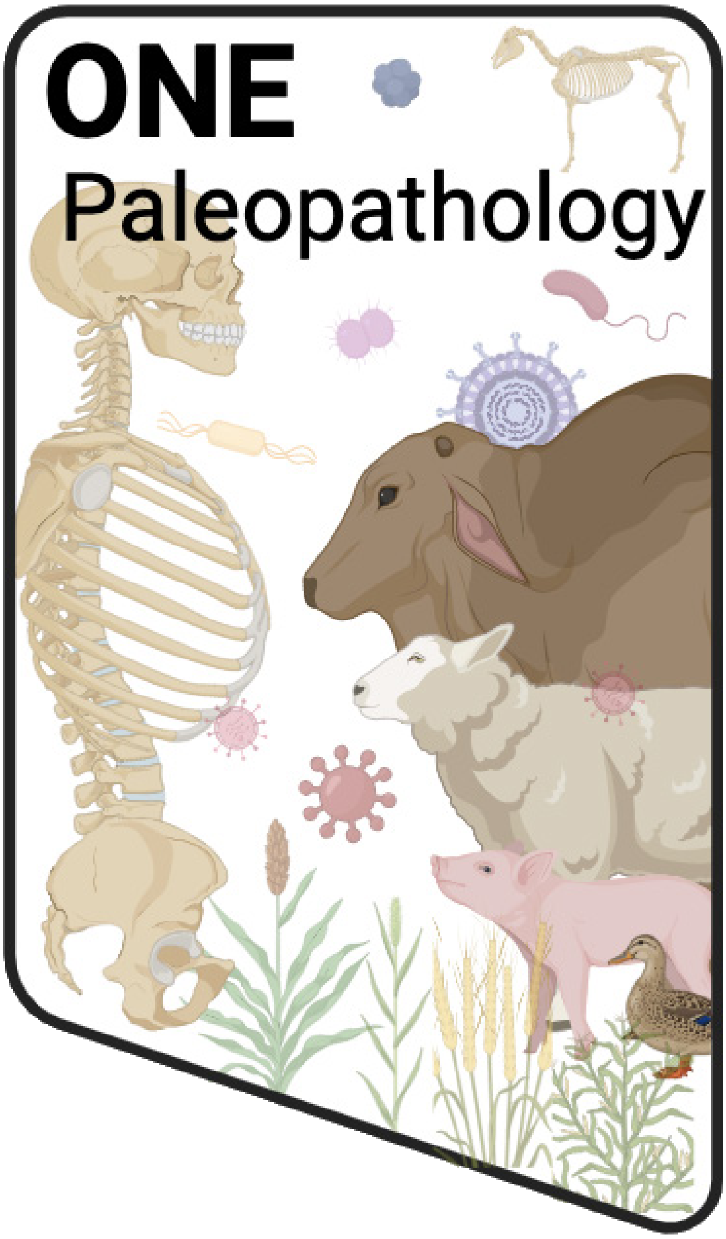

ONE Paleopathology Workshop

Schedule

AUGUST 4TH

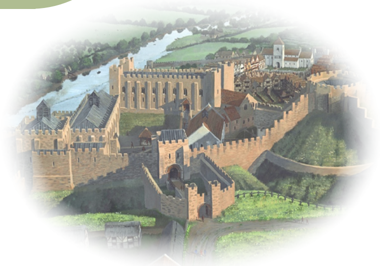

6:30-9:00 PM — **OPENING RECEPTION**
Bishops Dining Hall, Castle

AUGUST 5TH: MORNING

Calman 407, All Participants Together

9:00 AM — **WELCOME, HISTORY OF CONCEPT, OUTLINE OF CONFERENCE**
Buikstra & Uhl

9:30 AM — **VISION, OUTCOMES, AND ASSESSMENT**
Schug

9:45 AM — **METHODS**
Imaging, AI, Epigenetics: Van Schaik
Modelling: Maasch

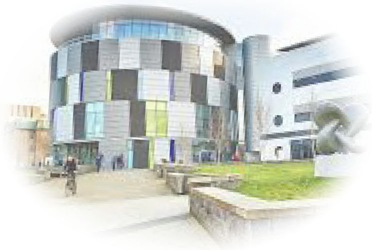

10:45 AM — **COFFEE BREAK**
Calman 406

11:15 AM — **INTERDISCIPLINARITY**
Disease Ecology: Gottdenker
Ecology of Historical Epidemics: Webster

11:45 AM — **EXAMPLES OF SUCCESSFUL INTERDISCIPLINARY PROJECTS**
Veterinary Sports Medicine: Thomas & Uhl
Public Health: Tomasto
Disease Risk: O'Donnell

12:15 PM — **DISCUSSION**

12:45 PM — **LUNCH & INFORMAL DISCUSSIONS**
Calman 406

AUGUST 5TH: AFTERNOON

Breakout Groups: Open Discussions

2:00 PM

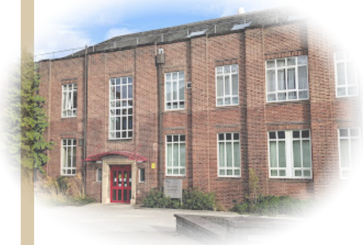

DISEASE SPILLOVER CLUSTER
Calman 407

Facilitators:
2:00-3:30 pm: Buikstra, Stone
2:00 pm: Blevins
2:45 pm: Wissler, Zuckerman

ENVIRONMENTAL TOXICITY & HEALTH
Dawson 216

Facilitators:
Marques, O'Donnell

CLIMATE: ENSO
Dawson 104

Facilitators:
Sandweiss, Snyder

AUGUST 5TH: AFTERNOON

Continued

3:30 PM — **COFFEE BREAK**
Calman 406

4:00 PM

MALARIA
Dawson 217
Facilitators: Littleton, Wilson

ENVIRONMENTAL TOXICITY & HEALTH
Dawson 216 (continued)
Facilitators: Marques, O'Donnell

CLIMATE: ENSO
Dawson 104 (continued)
Facilitators: Sandweiss, Snyder

ANIMALS AS SENTINELS, NOT VILLAINS
Calman 407
Facilitators: Nelson, Thomas

SYNDEMICS & INEQUALITY
Dawson 210
Facilitators: Gowland, Schaik

7:00-8:30 PM — **DINNER**
Bishops Dining Hall, Castle

AUGUST 6TH: MORNING

Breakout Groups: Focused Discussions: Goals
Goals will involve reaching consensus on contents of Working Paper, including plans for specialist, interdisciplinary, and non-specialist publications and other forms of outreach.

9:00 AM

MALARIA
Dawson 217
Facilitators: Littleton, Wilson

ENVIRONMENTAL TOXICITY & HEALTH
Dawson 216
Facilitators: Marques, O'Donnell

CLIMATE: ENSO
Dawson 104
Facilitators: Sandweiss, Snyder

ANIMALS AS SENTINELS, NOT VILLAINS
Calman 407
Facilitators: Nelson, Thomas

SYNDEMICS & INEQUALITY
Dawson 210
Facilitators: Gowland, Schaik

ONE Paleopathology Workshop

Schedule

AUGUST 6TH: MORNING

Continued

10:45 AM — **COFFEE BREAK**
Calman 406

11:15 AM — **OUTREACH ACTIONS**
Calman 407, All Participants Together
Outreach ACTIONS – Schug
11:30 am ***Virtual****: Louie Sandys, Cambridge U Press Research Directions*
12:00 pm: Cynthia Beall

12:15 PM — **LUNCH**
Calman 406

AUGUST 6TH: AFTERNOON

Calman 407, All Participants Together to Share Results and Prioritize future activities.

1:30 PM — **CLOSING REPORTS**

3:30 PM — **COFFEE BREAK**
Calman 406

4:00 PM — **INFORMAL DISCUSSIONS & TOUR OF PALEOPATHOLOGICAL COLLECTIONS**
Calman 407, Gowland

6:15 PM — **CLOSING RECEPTION**
Cathedral

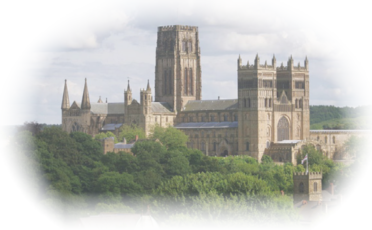

Use this QR code to access the ONE Paleopathology Workshop Dropbox folder anytime!
