# Supplementary material for: Assessing the effectiveness of the one paleopathology workshop
Source: Evol Med Public Health. 2026 Jan 6;14(1):1–10. doi: 10.1093/emph/eoaf041 (PMC12874872; doi:10.1093/emph/eoaf041)
Supplement: Supplemental_File_C_-_Pre-workshop_Questions_eoaf041 [file supplemental_file_c_-_pre-workshop_questions_eoaf041.pdf]

Pre-Workshop Discussion Questions Provided to Participants

Animals as Sentinels, Not Villains:

- How best can we frame questions that establish the significant role animal paleopathology should be playing in ONE Paleopathology?
- Given the limitations imposed by the fragmentary and incomplete archaeological record, what other sources of information help us develop a ONE Paleopathology approach to big picture questions.
- How best can we employ aDNA to enhance animal paleopathology in ONE Paleopathology?
- How can we more effectively understand the link between early domestication processes and associated zoonotic disease risks?
- Under what conditions do diseases jump to animals and become pathogenic, e.g. *Burkholderia mellei*?
- What roles do modelling and general ecological theories have in ONE Paleopathology?
- What would be research projects designed to illustrate the significance of the (nonhuman) animal component in ONE Paleopathology and synergism with human paleopathology?
- How can we more effectively understand the link between early domestication processes and associated zoonotic disease risks?

Climate Change (ENSO events in Peru):

- What are currently the best methods for estimating the **timing** of ENSO events?
  - Could cemental annuli in some animals (including humans) record ENSO events?
- How precise can we be about measuring time for **short-and-long-term** events?
- How should we think about human resilience?
  - Behavioral responses?
  - Health-related impacts?
  - Should we explicitly emphasize theories and if so, which one?
  - How should be best integrate non-human information as cues to impact and resiliency.
- How best can we generate research designs that will maximize local data along with that for cross-region comparisons?
- What role might the modeling of Chagas Disease have for our deliberations?
- How should be best integrate non-human information as cues to impact and resiliency.
- Is there are role for traditional knowledge in this endeavor?
- How best can we generate research designs that will maximize all relevant sources of data?

- What should be our explicit plans for future research (basic; transdisciplinary) and outreach (to whom—policy-makers, public communities, colleagues-- and in what form)

Environmental Toxicity:

- Which environmental toxicants are of interest?
    - Heavy Metals (which ones)
    - Radon
    - Microplastics
- How best do we investigate dated archaeological contexts relationship to toxicity?
- How do we interact with communities living in regions known to have ancient toxicants?
- How do we integrate the industrial shift to batteries (lithium, zinc) into our plans for research and outreach?
- How best do we integrate traditional knowledge?
- How best do we integrate knowledge of impact on animals into this form of study?
- What can we gain from the field of medical geology?
- How have humans adapted to toxic environments, biologically and socially? Mario Apata and Bernardo Arriaza have been studying the genetic adaptation to arsenic among ancient peoples of the Azapa Valley in northern Chile.
- How best can we generate research designs that will maximize all relevant sources of data?
- What should be our explicit plans for future research (basic; transdisciplinary) and outreach (to whom—policy-makers, public communities, colleagues-- and in what form)

Malaria:

- What approaches are we each taking?
- How can we work together to generate further excitement in our projects?
- Are collaborative research proposals possible?
- What skeletal evidence is reliable in evaluating malaria and its impact? Can we confidently identify presence? Prevalence? Impact?
- How do we best interpret archival evidence?
- How do we communicate the probable future for malaria in a warming world?
- Is traditional knowledge and its practitioners part of our portfolio?

Syndemics and Inequality:

- What is the best working definition for "syndemics" in a bioarchaeological (or ONE Paleopathological) perspective?
- Syndemics emphasized the complexity of disease interactions with contextual variables. This implies a local, small-scale approach. HOW do we then develop

meaningful generalizations of broad relevance? Is Singer et al.'s (2021:8; from Perry and Gowland, 2022:37) concept of "syndemogeny" help solve this problem?

- How can we integrate animal paleopathology into syndemics? Please provide examples.
- How can syndemics in past contexts deliver a transdisciplinary result? Examples?
