# Supplementary material for: Assessing the effectiveness of the one paleopathology workshop
Source: Evol Med Public Health. 2026 Jan 6;14(1):1–10. doi: 10.1093/emph/eoaf041 (PMC12874872; doi:10.1093/emph/eoaf041)
Supplement: Supplemental_File_D_-_Post-Workshop_Survey_eoaf041 [file supplemental_file_d_-_post-workshop_survey_eoaf041.pdf]

ONE Paleopathology Deliverables

Please use the printed version of this form to guide your discussions. However, we would appreciate it if you would fill out a digital version of this form for the facilitors and organizers. Below you will find a QR code that you can scan with your phone to take you to the digital form. Thanks!

1. Email *

____

QR code to access the digital version of this form

2. Name

3. Collaborative role

Mark only one oval.

- Facilitator
- Participant
- Student Volunteer

4. Breakout Group

Check all that apply.

- [ ] Disease Spillover Cluster
- [ ] Malaria
- [ ] Syndemics and Inequality
- [ ] Animals as Sentinels, not Villains
- [ ] Environmental Toxicity and Health
- [ ] Climate: ENSO

New Collaboration Description

Next, please describe your new collaborative ideas in the spaces provided. We are specifically interested in how this research is interdisciplinary, who is collaborating, and how the collaboration will generate proposed research

5. Working Title of New Collaboration:

6. Who is collaborating on this project?

7. Please briefly describe your new collaboration, what is the goal of the research?

____

____

____

____

____

8. What kinds of resources or expertise would you need to forward this new collaboration? How do you plan on acquire these resources or make connections to fill the needs of different gaps in expertise?

____

9. Which of the grants discussed are you interested in submitting proposals for? (pictures have the grant synopsis)

Check all that apply.

- [ ] NSF: Accelerating Research through International Network-to-Network Collaborations (AccelNet)
- [ ] NSF: Biodiversity on a Changing Planet (BoCP)
- [ ] Build and Broaden 3.0 (B2 3.0)
- [ ] NSF: Organismal Response to Climate Change (ORCC)
- [ ] Incorporating Human Behavior in Epidemiological Models (IHBEM)
- [ ] Climate Change and Human Health Seed Grants
- [ ] Wenner-Gren

10. Are there additional grants that you would be interested in submitting for? Or are there other grants not discussed that other participants might be interested in?

11. What are the expected publication venues for the proposed collaboration? (e.g. the Journal of Evolutionary Medicine, CUP ONE Health, Frontiers in Environmental Archaeology, or Frontiers in Veterinary Medicine)

12. The workshop organizers are planning a high-impact publication of a summary of this workshop. Are there high-impact venues (i.e. PNAS, Nature, etc) that you think the proposed collaboration might aim for?

Outreach to various audiences

The ONE Paleopathology Workshop wants to define specific and measurable goals for outreach activites related to these new collaborations that can raise awareness and present the unique opportunities of ONE Paleopathology such as defining toxicity baselines, the role of humans in disease propagation globally, or factors that promote resilience in the face of climate stressors.

13. What kinds of outreach activites does your collaboration aim to accomplish?

Mark only one oval.

- ( ) Shaping public policy
- ( ) Education
- ( ) Professional and collegial
- ( ) Other: ____________________

14. If you have specific outreach activities in mind, please describe them here:

____
____
____
____
____

This content is neither created nor endorsed by Google.

Google Forms
