# Supplementary material for: Assessing the effectiveness of the one paleopathology workshop
Source: Evol Med Public Health. 2026 Jan 6;14(1):1–10. doi: 10.1093/emph/eoaf041 (PMC12874872; doi:10.1093/emph/eoaf041)
Supplement: Supplemental_File_E_-_Outcomes_Assessment_Survey_eoaf041 [file supplemental_file_e_-_outcomes_assessment_survey_eoaf041.pdf]

One Paleopathology Outcomes Assessment

Dear One Paleopathology Workshop Colleagues,

It has been a year since we all got together in Durham to explore the themes of One Paleopathology! We are now assessing how effective the workshop was in terms of continued engagement with One Paleopathology. If we have sufficient participation in this survey, we will generate a multi-authored (those participating) manuscript focused on the development of One Paleopathology and the impact of tailored workshops on trans-disciplinarity. Please return the survey AS SOON AS POSSIBLE, but no later than **June 30** to be included in the publication!

* Indicates required question

1. Email *

2. Name:

Goals of the One Paleopathology Workshop

Our overall vision for this workshop was to:

1. expand the breadth and depth of the discipline of One Paleopathology by applying a holistic study of the past to modern public health, medical and veteranry science, and thereby
2. generate authentic, trans-disciplinary collaborations that embody the holistic and transformative ideas of One Paleopathology.

To determine the success of the workshop and how well we accomplished these big picture goals, we want to reflect on the changes brought on by the workshop to your understanding of One Paleopathology as a discipline and to your research agenda. Additionally, we want to assess the direct results of the workshop (i.e. research collabroations and products generated) as well as how the workshop changed your views on trans-disciplinary research.

One Paleopathology as a disipline

3. Has your understanding of One Paleopathology changed since attending this workshop? *

Mark only one oval.

- ( ) Yes
- ( ) No

4. Please reflect on how your understanding of One Paleopathology has changed/expanded since attending this conference?

____
____
____
____
____

5. What is your working definition of One Paleopathology?

____
____
____
____
____

6. How significant is the One Paleopathology concept?
   1.) for Paleopathology as a discipline, and

   2.) for trans-disciplinarity

   ____
   ____
   ____
   ____
   ____

7. Will you use the term “One Paleopathology”? Why or why not?

   ____
   ____
   ____
   ____
   ____

Collaborations generated and general impact on trans-disciplinary research

8. Next, we want to assess the direct results of the workshop. Please check any of the following statements that you feel apply. *

   As a direct result of the One PP Workshop, I:

   Check all that apply.

   - [ ] formed new research collaborations
   - [ ] planned or wrote new grant proposals
   - [ ] planned or wrote new articles or chapters
   - [ ] planned or gave new conference presentations
   - [ ] adjusted my course content or materials in the classroom
   - [ ] Other: ______________________________

9. From your response to the question above, can you expand on what you feel are direct results of the workshop?

10. How has your concept of interdisciplinary/transdisciplinary research changed as a result of this workshop?

11. Has a deeper understanding of One Paleopatholgy changed how you interact with paleopathology, anthropology, or teaching more broadly?

12. Other comments:

Thank you!!

Thank you for taking the time to fill out this assessment!

This content is neither created nor endorsed by Google.

Google Forms
